# Intercontinental genetic structure and gene flow in Dunlin (*Calidris alpina*), a potential vector of avian influenza

**DOI:** 10.1111/eva.12239

**Published:** 2015-01-28

**Authors:** Mark P Miller, Susan M Haig, Thomas D Mullins, Luzhang Ruan, Bruce Casler, Alexei Dondua, H River Gates, J Matthew Johnson, Steve Kendall, Pavel S Tomkovich, Diane Tracy, Olga P Valchuk, Richard B Lanctot

**Affiliations:** 1 U.S. Geological Survey, Forest and Rangeland Ecosystem Science CenterCorvallis, OR, USA; 2 School of Life Sciences and Food Engineering, Nanchang UniversityNanchang, China; 3 Izembek National Wildlife RefugeCold Bay, AK, USA; 4 Beringia National ParkProvidenia, Russia; 5 U.S. Fish and Wildlife Service, Migratory Bird ManagementAnchorage, AK, USA; 6 U.S. Fish and Wildlife Service, Arctic National Wildlife RefugeFairbanks, AK, USA; 7 Zoological Museum, Lomonosov Moscow State UniversityMoscow, Russia; 8 Anchor PointAK, USA; 9 Institute of Biology and Soil Science, Russian Academy of ScienceVladivostok, Russia

**Keywords:** *Calidris alpina*, Dunlin, genetic structure, highly pathogenic avian influenza, human disease, influenza A, migratory connectivity, migratory short-stopping

## Abstract

Waterfowl (Anseriformes) and shorebirds (Charadriiformes) are the most common wild vectors of influenza A viruses. Due to their migratory behavior, some may transmit disease over long distances. Migratory connectivity studies can link breeding and nonbreeding grounds while illustrating potential interactions among populations that may spread diseases. We investigated Dunlin (*Calidris alpina*), a shorebird with a subspecies (*C. a. arcticola*) that migrates from nonbreeding areas endemic to avian influenza in eastern Asia to breeding grounds in northern Alaska. Using microsatellites and mitochondrial DNA, we illustrate genetic structure among six subspecies: *C. a. arcticola*,*C. a. pacifica*,*C. a. hudsonia*,*C. a. sakhalina*,*C. a. kistchinski*, and *C. a. actites*. We demonstrate that mitochondrial DNA can help distinguish *C. a. arcticola* on the Asian nonbreeding grounds with >70% accuracy depending on their relative abundance, indicating that genetics can help determine whether *C. a. arcticola* occurs where they may be exposed to highly pathogenic avian influenza (HPAI) during outbreaks. Our data reveal asymmetric intercontinental gene flow, with some *C. a. arcticola* short-stopping migration to breed with *C. a. pacifica* in western Alaska. Because *C. a. pacifica* migrates along the Pacific Coast of North America, interactions between these subspecies and other taxa provide route for transmission of HPAI into other parts of North America.

## Introduction

Birds are primary reservoirs for all known influenza A virus subtypes (Webster et al. 1992). In particular, waterfowl (Anseriformes) and shorebirds (Charadriiformes) are the most common wild vectors (Olsen et al. 2006). Infected birds generally harbor low-pathogenic avian influenza (AI) strains; however, outbreaks of highly pathogenic avian influenza strains (HPAI) such as the H5N1 and H7N9 subtypes are becoming more common, especially in South-East Asia (Chen et al. 2004, 2006; Li et al. 2004; Ferguson et al. 2005; Gao et al. 2013; Uyeki and Cox 2013). Concerns surrounding the spread of HPAI exist, particularly as mediated through avian vectors given the long distance seasonal migratory behavior of many virus hosts (Kilpatrick et al. 2006). Although most migratory movements occur within continents, intercontinental migration can also occur. For example, up to three million birds and thousands of infected individuals cross the Bering Strait from Asia into Alaska each year (Winker and Gibson 2010).

The likelihood that an individual bird species may contribute to the intercontinental spread of avian influenza depends in part on the details of its seasonal migratory patterns. Thus, migratory connectivity studies of birds can be used to define important migratory pathways and identify the population of origin of individuals at all stages of the annual cycle (Webster et al. 2002). Such studies take on new importance in the age of widespread disease transfer by birds (e.g., Rappole et al. 2000; Ishiguro et al. 2005; Morshed et al. 2005; Fergus et al. 2006; Gilbert et al. 2006; Dusek et al. 2014). If the identity and origin of avian disease carriers can be determined and if their migratory pathways are understood, it may be possible to predict the next occurrence of a virulent disease near human population centers, implement precautionary measures to limit human–bird contact, and adopt practices to try to minimize the potential for further spread of the disease to other geographic regions.

**Figure 1:**
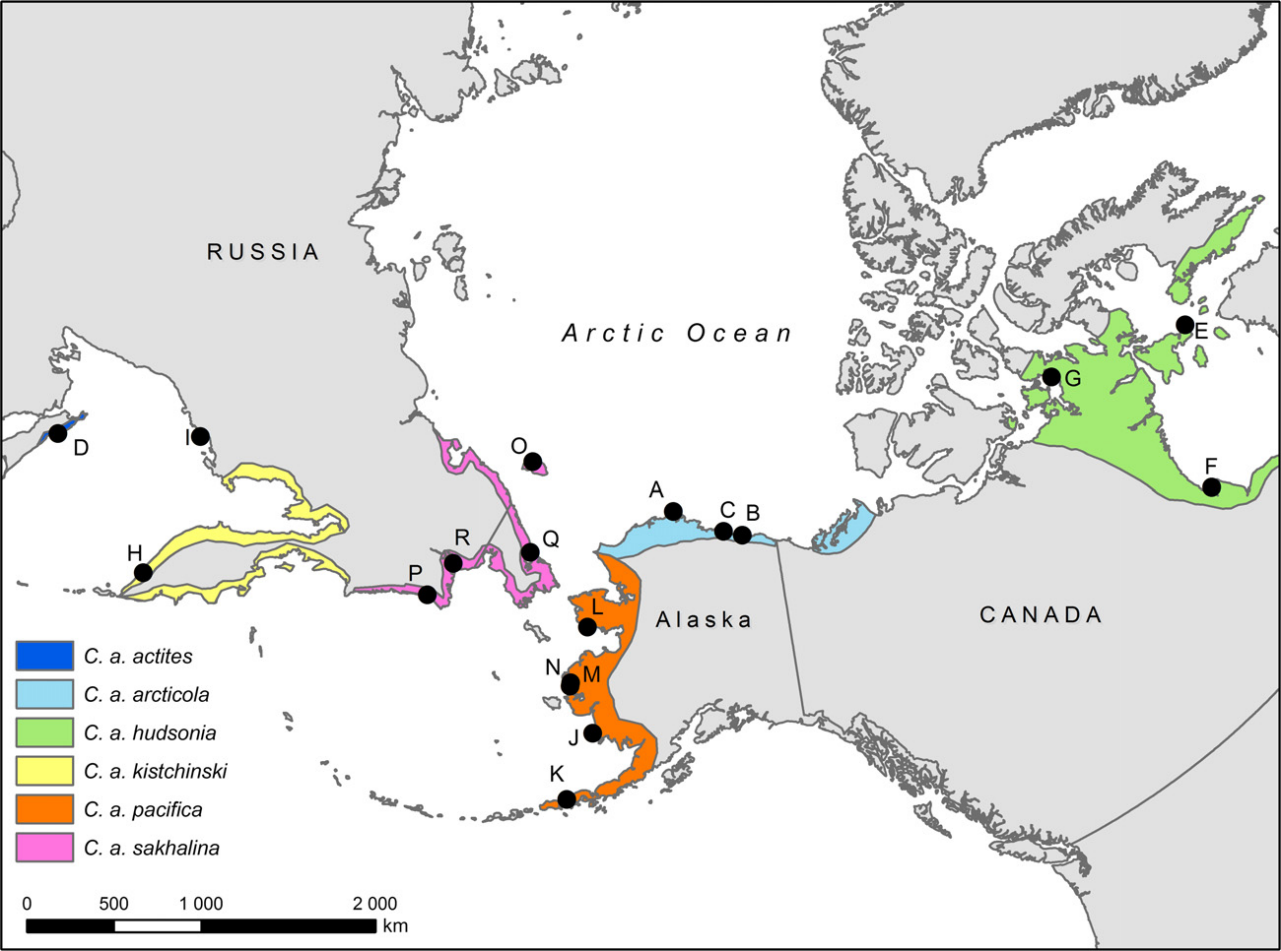
Breeding distribution of six subspecies of Dunlin (*Calidris alpina*) sampled for genetic analysis. Sites codes are congruent with those listed in Table1.

The Dunlin (*Calidris alpina*) is a circumpolar migratory shorebird that breeds throughout arctic and subarctic tundra regions and winters in the southern portion of the Northern Hemisphere (Del Hoyo et al. 1996). There are up to 11 described subspecies that show varying degrees of morphological variation (Greenwood 1986; Tomkovich 1986; Nechaev and Tomkovich 1987; Browning 1991; AOU 2013). These purported subspecies are believed to use separate breeding grounds, but their migratory flyways and nonbreeding areas may overlap (Warnock and Gill 1996; Lappo et al. 2012; Gill et al. 2013). Five subspecies of Dunlin breed in the East Asia and Alaska region known as Beringia (Fig.1): *Calidris alpina actites*,*C. a. kistchinski*, and *C. a. sakhalina* breed in the Russian Far East while *C. a. arcticola* and *C. a. pacifica* breed in Alaska (Warnock and Gill 1996; AOU 2013; Fig.1). A sixth North American subspecies exists (*C. a. hudsonia*), but breeds in central and eastern Canada and winters along the Atlantic Coast and Gulf of Mexico (Fernández et al. 2008). Potential interactions among the Beringia subspecies are complex: *C. a. pacifica* breeds in western Alaska and migrates south along the Pacific Coast of North America to winter in the western United States and Mexico (Fernández et al. 2008; Gill et al. 2013). *Calidris alpina arcticola* breeds in northern Alaska, but migrates across the Bering Strait to winter along the Pacific Coast of Asia where it potentially intermixes with the three East Asia subspecies (Fernández et al. 2008; Lanctot et al. 2009; Gill et al. 2013).

Dunlin were ranked the second-highest of 26 priority taxa to be routinely monitored for HPAI in Alaska when extensive sampling was initiated during the H5N1 HPAI outbreak in 2006 (U.S. Fish and Wildlife Service and U.S. Geological Survey 2007). The rankings were based on each taxon's distribution in Asia, proximity to locations where HPAI has been previously identified, general habitat use patterns, ease of sampling, and population size in Alaska (Alaska Interagency HPAI Bird Surveillance Working Group 2006; Ip et al. 2008). Dunlin ranked high primarily because they winter in areas where outbreaks of HPAI occur in Asia and because so many individuals (300 000–700 000 birds; Andres et al. 2012) migrate from Asia to Alaska each year. Dunlin are also highly susceptible to HPAI H5N1 (Hall et al. 2011). Mortality is likely common among infected juveniles (Hall et al. 2011), but infected adults may survive and transmit viruses. Surveys of wild-caught Dunlin in Alaska between 2006 and 2007 revealed that 0.22% were positive for AI based on RT-PCR analyses of cloacal swabs or fecal samples (Ip et al. 2008), indicating that active shedding of AI viruses was occurring at the time of sampling. This value likely underestimates the true infection rate, as Hall et al. (2011) found that RT-PCR detection of H5N1 in experimental challenges was longer lasting and more consistent from oropharyngeal samples as opposed to cloacal samples. Furthermore, Pearce et al. (2012) found that 2.6% of Dunlin sampled in Alaska during the late summer of 2010 demonstrated evidence for prior AI exposure based on serologic assays. While actual numbers are likely to vary substantially from year to year based on the dynamics of viral outbreaks in Asia, these studies nominally suggest that between 1540 and 18 200 (based on estimated population sizes) infected Dunlin could be in Alaska in any given year. Collectively, this information indicates that *C. a. arcticola* is an important subspecies to consider when evaluating potential routes and mechanisms by which Asian influenza strains can be transmitted to North America.

Although all Dunlin subspecies show some phenotypic variation (Tomkovich 1986; Nechaev and Tomkovich 1987; Browning 1991), it is difficult to separate them outside of the breeding grounds using commonly employed morphological characters such as plumage or culmen, head, wing, and tarsus measurements (Warnock and Gill 1996; Wennerberg et al. 1999; but see Gates et al. 2013). This is particularly true in eastern Asia where four subspecies are thought to intermix during the nonbreeding season (Lanctot et al. 2009; Gates et al. 2013). In these circumstances, hypervariable molecular markers and DNA sequences may be useful for illuminating patterns of population connectivity and movements of individuals throughout the annual cycle (Haig et al. 2011). We therefore used mitochondrial DNA sequences (mtDNA) from the cytochrome *b* gene and control region along with eight nuclear microsatellite loci to address multiple questions associated with the differentiation of Dunlin subspecies and the extent of gene flow and interactions among groups from Asia and North America. (i) Do genetic data provide evidence for differentiation among Dunlin subspecies and breeding populations from the region? While prior work has examined phylogeographic patterns in the Northern Hemisphere, most studies were based on small sample sizes and had limited (or no) sampling within Beringia-associated subspecies (e.g., Wenink and Baker 1996; Wenink et al. 1996; Wennerberg et al. 1999; Marthinsen et al. 2007). (ii) Does genetic differentiation among subspecies provide a basis for the probabilistic identification of subspecies where they co-occur (*sensu* Patten and Unitt 2002)? In particular, we were interested in determining whether genetic data can distinguish *C. a. arcticola* from the three other Dunlin subspecies that winter in East Asia (*C. a. actites, C. a. kistchinski*, and *C. a. sakhalina*). If distinguishable, then nonbreeding populations of *C. a. arcticola* could be more easily identified, leading to a better understanding of the likelihood of this subspecies becoming infected and transmitting HPAI into North America. (iii) Can genetic data characterize the extent of gene flow and interaction among the three proximate Beringian subspecies (*C. a. sakhalina*,*C. a. arcticola*, and *C. a. pacifica*)? Given the geographic locations of their breeding ranges (Fig.1), opportunities for gene flow among subspecies may occur. Furthermore, a portion of the *C. a. arcticola* and *C. a. pacifica* populations intermix during postbreeding staging in western Alaska (Gill et al. 2013), but the extent of gene flow between these groups is not well known. If gene flow is extensive, then the data may point to greater-than-expected interactions between these two subspecies. Because *C. a. pacifica* winters along the Pacific Coast of North America, interactions with *C. a. arcticola* during the breeding or postbreeding season may increase the risk of transmission of Asian influenza strains from Alaska into other parts of North America.

## Materials and methods

### Sample collection and molecular methods

We collected 370 Dunlin blood or tissue samples from 18 breeding areas during the 2003 to 2009 breeding seasons (Fig.1, Table1). Samples included putative representatives from the five subspecies that inhabit eastern Asia and Alaska (*C. a. actites*,*C. a. kistchinski*,*C. a. sakhalina*,*C. a. pacifica*, and *C. a. arcticola*) and were our primary focus for this study. However, we also included samples from three *C. a. hudsonia* breeding populations in eastern North America to help provide greater genetic and spatial context to our analyses. Individual birds were captured with bownets at nest sites (most subspecies) or lethally collected (*C. a. kistchinski* samples) on breeding territories. Live-captured birds had up to 0.3 mL of blood collected into a heparinized tube via brachial puncture with a 26- to 27.5-gauge needle. Additional breeding season tissues were obtained from the University of Washington Burke Museum to augment the Russian populations (UWBM Accession Numbers 43910, 44120, 44121, 51684, 51687, 51693, 51694, 51695, and 69903). Blood or tissue samples were preserved in Longmire buffer (Longmire et al. 1997) until used for genetic analyses.

**Table 1:** Sample sizes and locations of six subspecies of Dunlin (*Calidris alpina*) sampled for microsatellites (*n* = 370) and mtDNA (*n* = 234). Locations are indicated by site code on Fig.1

Subspecies	Location (site code)	Latitude	Longitude	*N* (microsats)	*N* (mtDNA)
*arcticola*	Barrow, AK, USA (A)	71.27	−156.53	87	32
	Canning, AK, USA (B)	70.10	−145.85	34	15
	Prudhoe Bay, AK, USA (C)	70.35	−148.64	23	13
*actites*	Schiavo Bay, Sakhalin, Russia (D)	52.55	+143.30	23	23
*hudsonia*	Nunavut, NU, Canada (E)	63.97	−80.28	3	3
	Churchill, MB, Canada (F)	58.74	−94.07	10	10
	Rasmussen, NU, Canada (G)	69.02	−93.85	3	3
*kistchinski*	Kamchatka, Russia (H)	52.81	+156.42	30	25
	Magadanskaya Oblast, Russia (I)	59.38	+149.07	12	5
*pacifica*	Platinum, AK, USA (J)	59.02	−161.82	8	7
	Cold Bay, AK, USA (K)	55.24	−162.84	25	21
	Nome, AK, USA (L)	64.45	−164.93	5	4
	Kanaryarmiut, AK, USA (M)	61.36	−165.15	8	8
	Manokinak, AK, USA (*N*)	61.19	−165.10	30	11
*sakhalina*	Wrangel, Russia (O)	71.41	−179.67	20	16
	Meinopylgino, Chukotka, Russia (P)	62.55	+177.08	11	10
	Belyaka Spit, Chukota, Russia (Q)	67.15	−174.68	22	16
	Anadyr, Chukota, Russia (R)	64.70	+177.63	16	12

DNA was extracted as described in Haig et al. (2004). We used polymerase chain reaction (PCR) to amplify partial sequences of the mitochondrial cytochrome *b* gene (cyt *b*) and control region (D-loop) in 234 samples (Table1). Primer pairs, including L14996-H15646 (http://people.bu.edu/msoren/primers.html, accessed 15 January 2015) and TS96L-TS778H (Wenink et al. 1994), were used to amplify the mitochondrial *cyt* b and D-loop sequences, respectively. All primer sequences and annealing temperatures are shown in Appendix A. PCR amplifications were performed in 20 μL reactions containing 2.5 mm MgCl_2_, 1 μm of primers, 100 μm of each dNTP, 1× PCR buffer (Perkin Elmer, Waltham, MA, USA), and 1 U AmpliTaq Gold DNA polymerase (Perkin Elmer). Thermal-cycling parameters included initial denaturation at 94°C followed by 35 cycles of denaturing at 94°C (30 s), the annealing temperature listed in Appendix A (30 s), and extension at 72°C (60 s). PCR products were bidirectionally sequenced with BigDye® Terminator 3.1 Cycle Sequencing chemistry (Life Technologies, Grand Island, NY, USA) and resolved on an ABI 3730 automated DNA sequencer, with resulting chromatograms aligned, edited, and trimmed using the program SeqMan ver. 8.0.2 (DNAStar Inc., Madison, WI, USA). The final 1112-bp alignment contained concatenated sequences from each individual and included 633 bp of *cyt* b and 479 bp from the D-loop.

Nuclear microsatellite genotypes were obtained at eight loci for 370 individuals (Table1; Appendix A). We obtained primers for loci CALP2 and 4A11 from Wennerberg (2001a), and for loci Cme2, Cme10, and Cme12 from van Treuren et al. (1999), whereas loci D25, D26, and D110 were characterized *de novo* for this specific investigation during an Illumina GAIIx Genome Analyzer paired-end 80 run (*sensu* Jennings et al. 2011). Library construction followed recommended Illumina protocols with the exception that index sequencing ‘bar-coded’ adapters (Craig et al. 2008; Cronn et al. 2008) were substituted for standard paired-end adapters. Primer sequences and annealing temperatures for all microsatellites are provided in Appendix A. PCRs were performed in a 10 μL reaction volume with the following reagent concentrations: 1× PCR buffer (Promega Inc., Madison, WI, USA), 0.5 μm of each primer, 2.5 mm MgCl_2_, 100 μm of each dNTP, and 1 U Taq DNA polymerase (Promega, Inc.). Thermal-cycling parameters included 2 min denaturation at 93°C, followed by 30 cycles of 30 s at 93°C, 30 s at the appropriate annealing temperature, and elongation at 72°C for 1 min. Amplification products were analyzed on an ABI 3100 capillary DNA automated sequencer. ABI GENESCAN software was used to size fragments based on internal lane standard GeneScan 500 [Rox]. ABI GENEMAPPER software was used to score alleles sizes.

### Differentiation among subspecies

We characterized the mitochondrial and microsatellite data to provide heuristic indicators of differences among subspecies. For the mtDNA data, we used FaBox (Villesen 2007) to identify unique haplotypes in the data set and create tables reflecting haplotype frequencies and shared haplotypes among groups. ARLEQUIN version 3.1 (Excoffier et al. 2005) was used to quantify gene diversity (*H*) and nucleotide diversity (*π*) in mtDNA data within each subspecies. Tables documenting microsatellite allele frequency variation among subspecies were created using CONVERT (Glaubitz 2004). Likewise, program GDA version 1.1 (Lewis and Zaykin 2002) was used to calculate allelic richness and observed and expected heterozygosity (H_O_ and H_E_, respectively). HP-Rare (Kalinowski 2005) was used to obtain rarefied estimates of allelic richness that accounted for differences in sample size.

We used phylogenetic analyses to examine differentiation of subspecies based on the mtDNA data. The program PhyML 3.0 (Guindon et al. 2010) was used to infer phylogenetic relationships among haplotypes using the maximum-likelihood (ML) criterion. The best-fit nucleotide substitution model was identified using jModeltest2 (Darriba et al. 2012). One thousand bootstrap replicates were used to evaluate clade support. Bayesian phylogenetic analyses were performed using MRBAYES version 3.1.2 (Huelsenbeck and Ronquist 2001), where four concurrent chains were run for 6 × 10^6^ generations. Trees were sampled every 2000 generations and ‘burn in’ included the initial 25% of samples. jModeltest 2 was also used to identify nucleotide substitution models for Bayesian analyses, but was restricted to the subset of models supported by MRBAYES when performing model selection. Resulting phylogenetic trees from both analyses were visualized and annotated using MEGA 5.2 (Tamura et al. 2011).

We used STRUCTURE version 2.2.3 (Pritchard et al. 2000) to analyze the microsatellite data to identify the number of genetic clusters and to probabilistically assign each analyzed individual to one of the identified clusters. Analyses assumed numbers of clusters (*K*) ranging from one through seven and were based on the uncorrelated allele frequency model and no admixture. Ten replicate analyses were performed for each value of *K* with each replicate using an initial 10^6^ burn-in steps followed by 10^7^ analysis replicates. We evaluated the outcome of analyses in two different ways: by identifying the value of *K* that produced the highest average likelihood score over replicates and through the use of the Δ*K* procedure of Evanno et al. (2005). In both cases, results were summarized over replicates using the program CLUMPP (Jakobsson and Rosenberg 2007). Prior to all microsatellite analyses, we used GDA version 1.1 (Lewis and Zaykin 2002) to identify deviations from Hardy–Weinberg genotypic proportions and test for linkage disequilibrium between pairs of loci within each subspecies. Composite test results for Hardy–Weinberg disequilibrium within each subspecies were obtained by combining *P*-values from locus-specific analyses using the *Z*-transform test (Whitlock 2005).

ARLEQUIN was used to perform an analysis of molecular variance (amova; Excoffier et al. 1992) and quantify genetic structure among Dunlin subspecies. In this analysis, *Φ*st (for mtDNA), *F*_ST_, and *R*_ST_ (both for microsatellite data, the latter assuming a strict stepwise mutation; Slatkin 1995) were calculated to determine the overall and pairwise levels of differentiation among different subspecies. *P*-values associated with these statistics were obtained using 10 000 randomization replicates.

### Distinguishing *C. a. arcticola* from other subspecies that winter in Asia

Results from STRUCTURE analyses (described above) were further evaluated to determine whether the microsatellite data could be used to probabilistically distinguish among Dunlin subspecies that winter in Asia. If STRUCTURE identified more than one cluster, then assignment values for individuals within each cluster may facilitate accurate subspecific diagnoses of individual birds from mixed groups on the nonbreeding grounds. We also used the individual assignment approach encapsulated in GeneClass2 (Piry et al. 2004), where we determined whether birds could be assigned to one of the predefined Dunlin subspecies with a high degree of confidence. Analyses used the Bayesian computation criterion of Rannala and Mountain (1997) and probability computations as described in Cornuet et al. (1999) using 10 000 simulated individuals. After analyses, we determined the proportion of individuals that were correctly reassigned to their respective subspecies and the average probability associated with correct assignments.

**Figure 2:**
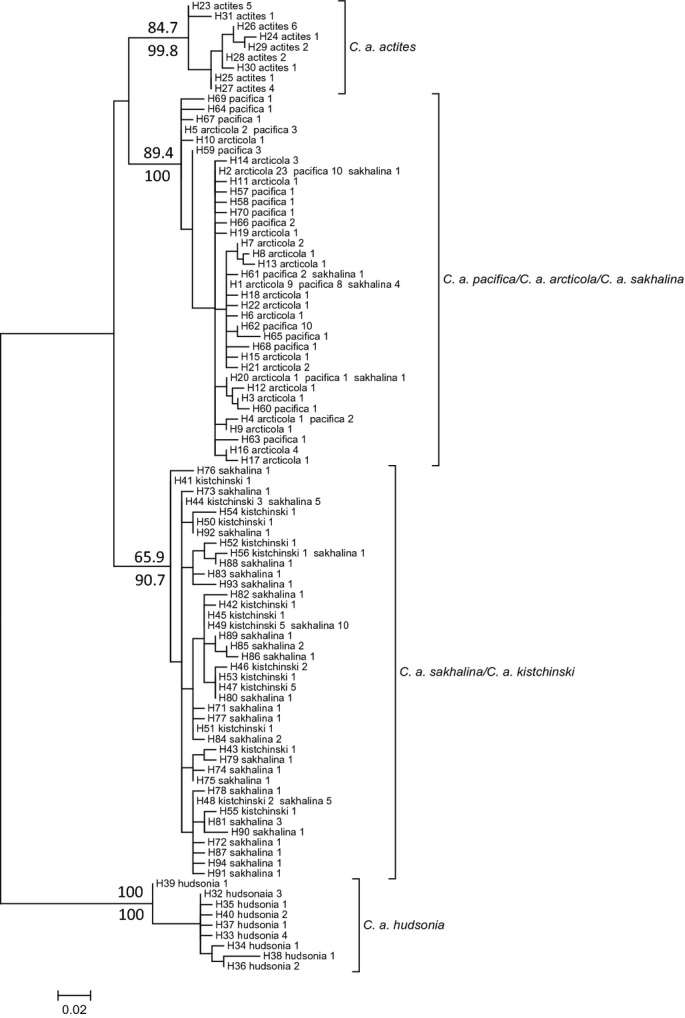
Unrooted maximum-likelihood (ML) tree generated from 94 mitochondrial DNA haplotypes detected in six subspecies of Dunlin (*Calidris alpina*). Labels at the terminus of each branch provide information on haplotype codes (Appendix B) and the number of individuals from each subspecies that possessed a given haplotype. Branch support values for four major clades of interest are indicated (above branch: bootstrap values from ML analyses; below branch: posterior probabilities from Bayesian analyses).

The diagnostic utility of the mtDNA data was also evaluated. Results from the phylogenetic analyses initially suggested that our mtDNA could be used to distinguish *C. a. arcticola* from other subspecies that winter in Asia (see Results and Discussion). Specifically, haplotypes from *C. a. arcticola* and *C. a. pacifica* (hereafter referred to as clade I haplotypes) formed a clade that was largely distinct from haplotypes detected in the Asian subspecies *C. a. kistchinski*,*C. a. sakhalina*, and *C. a. actites* (see Results and Fig.2). The sole exception to this pattern was the detection of seven *C. a. sakhalina* individuals that possessed clade I haplotypes (Fig.2). Therefore, to more formally quantify the diagnostic potential of the mtDNA, we applied a simple formulation of Bayes' theorem (Sokal and Rohlf 1995) to estimate *P*(*arcticola*|I): the probability that an individual sampled on the nonbreeding grounds with a haplotype from clade I is actually *C. a. arcticola* rather than *C. a. sakhalina*. The probability is calculated as 

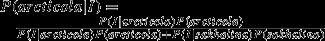
1 and relies on the following quantities: the probability of detecting clade I haplotypes in *C. a. arcticola*:*P*(I|*arcticola*) = 60/60 = 1.0; the probability of detecting clade I haplotypes in *C. a. sakhalina*:*P*(I|*sakhalina*) = 7/54 = 0.13; the probability of selecting a bird that is *C. a. arcticola*:*P*(a*rcticola*); and the probability of selecting a bird that is *C. a. sakhalina*:*P*(*sakhalina*) = 1 − *P*(*arcticola*). *Calidris alpina arcticola* and *C. a. sakhalina* are believed to use similar areas during the winter, primarily Japan, coastal mainland China, Taiwan, and South Korea (Lanctot et al. 2009; Clements et al. 2013; Gill et al. 2013). Because *P*(*arcticola*) and *P*(*sakhalina*) reflect the probability of randomly selecting an individual from each subspecies, these quantities therefore depend on the abundance of each subspecies on the wintering grounds. Based on population estimates, there are 100 000 to 1 000 000 *C. a. sakhalina* individuals (Bamford et al. 2008), whereas 300 000 to 700 000 *C. a. arcticola* winter in East Asia (Andres et al. 2012). We therefore calculated *P*(*arcticola*|I) using the upper bound, lower bound, and approximate midpoint of each population size estimate in calculations.

### Quantifying gene flow among Beringian subspecies

We used MIGRATE-N version 3.5.1 (Beerli and Palczewski 2010) to obtain Bayesian estimates of mutation-scaled effective population sizes and asymmetric migration rates among the three proximate Beringian subspecies (*C. a. sakhalina, C. a. arcticola*, and *C. a. pacifica*) that were most likely to exhibit gene flow. Limiting our analyses to three subspecies substantially reduced the number of parameters that needed to be simultaneously estimated, thereby providing a more tractable computational problem with a greater likelihood of success relative to analysis of the full data set (analysis required estimation of three as opposed to six effective population size parameters and six rather than thirty gene flow parameters) (Beerli 2009). MIGRATE-N estimates long-term effective population sizes as *θ *= *xN*_e_*μ*, where *μ* is the mutation rate and *x* is an inheritance scaling factor that takes on values of 1 for mtDNA and 4 for codominant nuclear markers such as microsatellites. Long-term migration patterns are estimated over the time scales reflected by the set of sampled gene genealogies using the mutation-scaled quantity *M* = *m*/*μ*, where *m* is the proportion of immigrants. Note that the product of the parameter estimates divided by the scaling factor (*θ*M/x) provides a basis for estimating *N*_e_*m*, the effective number of immigrants into a population per generation.

Analysis parameter values and settings for MIGRATE-N were selected after preliminary exploratory analyses and with input from the program's developer (P. Beerli, personal communication). mtDNA analyses used the basic DNA sequence model, and priors for *θ* were specified as a uniform distribution with minimum and maximum values of 0 and 0.03, respectively. Uniform priors with minimum and maximum values of 0 and 10 000 were likewise specified for M. Two independent runs based on random starting trees were performed to ensure convergence and consistency of parameter estimates. Each run was based on 10^6^ recorded steps with a recording interval of 50 steps. Four concurrent chains were implemented during each run, with each chain using a static heating scheme based on temperature values of 1.0, 1.5, 3.0, and 10^5^. Microsatellite analyses were performed using the Brownian motion model. Lower and upper bounds for the uniform prior on *θ* were specified as 0 and 10.0, whereas uniform priors for M were bound by 0 and 500. Two completely independent runs using starting UPGMA trees were performed, with each run based on 20 concurrent chains with 1000 recording steps made at 100 step intervals. The same heating scheme used for the mtDNA was applied to the microsatellites.

## Results

### Differentiation among subspecies

We observed 78 variable sites within the concatenated 1112-bp *cyt* b and D-loop sequence alignment (41 variable sites from *cyt* b and 37 from D-loop), which resulted in 94 unique haplotypes among the 234 Dunlin specimens examined (Appendix B; GenBank accessions for D-loop: KP205084– KP205177; GenBank accessions for *cyt* b: KP205178– KP205271). At the subspecies level, lowest values of mitochondrial gene and nucleotide diversities (*H* and *π*; Table2) were found in *C. a. arcticola* (H = 0.830, *π *= 0.0015), while the highest values were detected in *C*. *a. sakhalina* (H = 0.953, *π *= 0.0058, Table2). Most haplotypes were restricted to a single subspecies (84 of 94; Appendix B).

**Table 2:** Genetic diversity in Dunlin (*Calidris alpina*)

Subspecies	Microsatellites				mtDNA		
	*N*	*A*	*H*_E_	*H*_O_	*N*	*H*	*π*
*arcticola*	144	6.00 (4.46)	0.543	0.497	60	0.830	0.0015
*actites*	23	3.88 (3.71)	0.47	0.462	23	0.870	0.0019
*hudsonia*	16	4.38 (4.38)	0.536	0.508	16	0.908	0.0023
*kistchinski*	42	5.50 (4.64)	0.574	0.568	30	0.931	0.0028
*pacifica*	76	6.13 (4.66)	0.551	0.512	51	0.900	0.0023
*sakhalina*	69	6.63 (5.15)	0.604	0.545	54	0.953	0.0058

*N*, sample size; *A*, allelic richness (rarefied estimates accounting for differences in sample size provided in parentheses); *H*_E_, expected heterozygosity; *H*_O_, observed heterozygosity; *H*, gene diversity; *π*, nucleotide diversity.

jModeltest2 identified the TrN+I+G model as most appropriate for ML analyses. The unrooted ML tree grouped the 94 unique haplotypes into four clades that included (1) a *C. a. actites* group, (2) a *C. a*. *hudsonia* group, (3) a *C. a. kistchinski*/*sakhalina* group, and (4) a group comprised primarily of *C. a. arcticola*/*pacifica* specimens (Fig.2). With the exception of the detection of four *C. a. arcticola*/*pacifica* haplotypes in seven *C. a. sakhalina* specimens, there was no additional evidence of haplotype sharing among groups (Fig.2 and Appendix B). jModeltest2 indicated that the HKY model was most appropriate of those supported by MRBAYES. Trees from Bayesian analyses showed clear signs of convergence across the four runs (scale reduction factor of estimated parameters ranged from 0.99 to 1.01; standard deviation of split frequencies = 0.0093) and were virtually indistinguishable from the ML tree. Consequently, only the ML tree is presented here (Fig.2).

There was highly significant differentiation among subspecies based on the mitochondrial data (*Φ*_ST_ = 0.773, *P* < 0.001, Table3A). All pairwise comparisons among subspecies were highly significant (Table3A). Consistent with the phylogenetic analysis (Fig.2), the lowest *Φ*_ST_ values were detected in the *C. a. pacifica*/*arcticola* contrast and the *C. a. sakhalina*/*kistchinski* contrast—the two subspecies pairs that were not phylogenetically distinct in our analyses.

**Table 3:** Pairwise and global estimates of *F*_ST_ for Dunlin (*Calidris alpina*) subspecies. *F*_ST_ values are shown below matrix diagonals while *P*-values are above matrix diagonals. (A) mtDNA; (B) microsatellite analyses; (C) microsatellite analyses assuming a stepwise mutational model

A. *Φ*_ST_ = 0.773, *P* < 0.001	*arcticola*	*actites*	*hudsonia*	*kistchinski*	*pacifica*	*sakhalina*
*C. a. arcticola*		<0.001	<0.001	<0.001	<0.001	<0.001
*C. a. actites*	0.858		<0.001	<0.001	<0.001	<0.001
*C. a. hudsonia*	0.942	0.92		<0.001	<0.001	<0.001
*C. a. kistchinski*	0.867	0.797	0.894		<0.001	0.009
*C. a. pacifica*	0.048	0.814	0.92	0.832		<0.001
*C. a. sakhalina*	0.713	0.622	0.807	0.071	0.676	

B. *F*_ST_ = 0.032, *P* = 0.001	*arcticola*	*actites*	*hudsonia*	*kistchinski*	*pacifica*	*sakhalina*
*C. a. arcticola*		<0.001	<0.001	0.005	0.001	<0.001
*C. a. actites*	0.095		<0.001	<0.001	<0.001	<0.001
*C. a. hudsonia*	0.062	0.126		<0.001	<0.001	<0.001
*C. a. kistchinski*	0.010	0.097	0.065		0.019	0.129
*C. a. pacifica*	0.009	0.130	0.081	0.008		<0.001
*C. a. sakhalina*	0.014	0.094	0.058	0.004	0.022	

C. *R*_ST_ = 0.039, *P* < 0.001	*arcticola*	*actites*	*hudsonia*	*kistchinski*	*pacifica*	*sakhalina*
*C. a. arcticola*		0.042	<0.001	0.380	<0.001	0.033
*C. a. actites*	0.025		<0.001	0.013	<0.001	0.002
*C. a. hudsonia*	0.163	0.264		0.001	<0.001	0.003
*C. a. kistchinski*	0.001	0.057	0.130		0.006	0.414
*C. a. pacifica*	0.039	0.100	0.110	0.031		0.012
*C. a. sakhalina*	0.012	0.067	0.085	0.000	0.019	

As with the mtDNA data, *C. a. sakhalina* demonstrated the highest microsatellite allelic richness and *H*_E_ values. However, the lowest microsatellite diversity was detected in *C. a. actites* (Table2). The microsatellites demonstrated no evidence for significant deviations from Hardy–Weinberg genotypic proportions after sequential Bonferroni corrections. Likewise, the 168 linkage disequilibrium tests performed (28 locus-pair analyses per subspecies * 6 subspecies) revealed only five significant results at the 0.05 level. These significant tests were detected across several subspecies (*C. a*. *actites*,*C. a. pacifica*, and *C. a. sakhalina*) and could have been observed by chance alone given the large number of individual tests that were performed.

The microsatellite analyses provided varying insights regarding genetic differentiation patterns in Dunlin. STRUCTURE suggested no evidence of differentiation among subspecies. Although the greatest average likelihood score was observed for the *K *=* *5 case and the Δ*K* procedure suggested that there were *K *=* *2 clusters, individual assignment probabilities to individual clusters were low and nearly uniform across clusters (Appendix C). This outcome indicates that the analysis procedure overestimated the true number of clusters and that subspecies-level subdivisions cannot be resolved with this analytical approach. In contrast, the global estimate of *F*_ST_ from the microsatellite data indicated that significant genetic structure existed (*F*_ST_ = 0.032, *P *<* *0.001) (Table3B). However, in comparison with the mitochondrial analysis, the microsatellite differentiation was generally small and reflected subtle differences in allele frequencies among subspecies (Appendix D). Most pairwise subspecific measures of differentiation were significant, with the exception of the comparison of *C. a. sakhalina* and *C. a. kistchinski* (*F*_ST_ = 0.004, *P* = 0.129) (Table3B). The pairwise *R*_ST_ values and their associated *P*-values were similar to those of *F*_ST_ estimate, with the added finding of nonsignificant differentiation between *C. a. arcticola* and *C. a. kistchinski* (Table3C).

### Distinguishing *C. a. arcticola* from other subspecies that winter in Asia

Our STRUCTURE analyses suggested that the microsatellites possessed little utility for diagnosing subspecies (Appendix C). The GeneClass2 assignment tests provided similar insights. In general, success of the assignment approach was poor, with only 128 (34.6%) of the 370 individuals successfully assigned to the correct subspecies and only 31 of the 144 *C. a. arcticola* specimens (21.5%) correctly assigned. The average assignment probability of a properly assigned *C. a. arcticola* was only 0.576, indicating that there was low confidence in the correct assignments that were observed.

By contrast, our application of Bayes' theorem indicated a greater potential for genetic identification of *C. a. arcticola* if mtDNA data were used. In this case, the probability of a correct identification depends in part on the relative population sizes of *C. a. arcticola* and *C. a. sakhalina* (eqn 1; Table4): the two subspecies that winter in Asia and that also can possess a type I haplotype. Using the upper and lower bounds of population size estimates for each subspecies, our calculations suggest that, under the extreme case where the ratio of *C. a. sakhalina* to *C. a. arcticola* is 1 000 000:300 000, the probability that a bird possessing a clade I haplotype is a *C. a. arcticola* individual is 0.698 (Table4). This probability increases to 0.885 when population sizes are assumed to be equal and is as high as 0.982 when the population size of *C. a. arcticola* is assumed to be the upper extent of its estimated range and *C. a. sakhalina* is assumed to be at the lower extent of its range (Table4).

**Table 4:** Outcomes of calculations to infer *P*(*arcticola*|I): the probability that a randomly selected nonbreeding bird in East Asia with a haplotype from the main *C. a. arcticola*/*pacifica* group (Fig.2) is actually *C. a. arcticola* as opposed to *C. a. sakhalina*. Calculations depend on the relative abundance of *C. a. arcticola* and *C. a. sakhalina* and are described in the Materials and methods (eqn 1). This table presents outcomes that evaluated upper, lower, and approximate midpoint population size estimates given by Bamford et al. (2008) and Andres et al. (2012)

Population estimate					
Total	*P*(*sakhalina*)	*P*(*arcticola*)	*P*(*arcticola*|I)	*C. a. sakhalina*	*C. a. arcticola*
100 000	300 000	400 000	0.250	0.750	0.958
100 000	700 000	800 000	0.125	0.875	0.982
1 000 000	300 000	1 300 000	0.769	0.231	0.698
1 000 000	700 000	1 700 000	0.588	0.412	0.843
500 000	500 000	1 000 000	0.500	0.500	0.885

### Gene flow among Beringian subspecies

Results of MIGRATE-N analyses were comparable between independent runs for each data set, indicating that convergence had occurred. The posterior distributions for each parameter were also well defined (Appendix E), thus facilitating the generation of point estimates and credibility intervals for each parameter (Table5). In general, gene flow estimates were low. However, the signature of asymmetric gene flow was present in both data sets, where *C. a. arcticola* was a source of migrants into both *C. a. pacifica* and *C. a. sakhalina*, but comparatively little gene flow occurred in the opposite direction. Migration from *C. a. arcticola* into *C. a. pacifica* was particularly pronounced, especially based on the results of the mtDNA analysis (*M*_*arcticola* → *pacifica*_ = 3583.3) relative to the microsatellite data (*M*_*arcticola* → *pacifica*_ = 26.83). Migration from *C. a. arcticola* into *C. a. sakhalina* (mtDNA: *M*_*arcticola* → *sakhalina*_ = 90.0; microsatellites: *M*_*arcticola* → *sakhalina*_ = 13.5) was also detected, albeit at lower levels than the rate into *C. a. pacifica* (Table5).

**Table 5:** Bayesian estimates of mutation-scaled effective population sizes (*θ*) and asymmetric migration rates (M) among the Dunlin subspecies *C. a. arcticola, C. a. pacifica*, and *C. a. sakhalina*. 95% credibility intervals are reported for each parameter, as is the derived parameter *N*_e_*m* reflecting the effective number of migrants per generation. See text for more details. Posterior distributions of estimated parameters are illustrated in Appendix E

	mtDNA			Microsatellites		
	2.5%	Mode	97.5%	2.5%	Mode	97.5%
*θ*_*arcticola*_	0.0026	0.0049	0.0088	0.0000	0.0367	0.2000
*θ*_*pacifica*_	0.0047	0.0096	0.0281	0.0000	0.0300	0.1930
*θ*_*sakhalina*_	0.0075	0.0125	0.0209	0.0000	0.0300	0.1930
*M*_*pacifica* → *arcticola*_ (*N*_e_*m*)	0.0	3.3 (0.016)	1313.3	0.000	8.500 (0.078)	17.000
*M*_*sakhalina* → *arcticola*_ (*N*_e_*m*)	0.0	3.3 (0.016)	206.7	0.000	3.500 (0.032)	11.667
*M*_*arcticola* → *pacifica*_ (*N*_e_*m*)	1586.7	3583.3 (34.4)	8320.0	10.000	26.833 (0.201)	44.330
*M*_*sakhalina* → *pacifica*_ (*N*_e_*m*)	0.0	3.3 (0.032)	453.3	0.000	7.500 (0.056)	16.333
*M*_*arcticola* → *sakhalina*_ (*N*_e_*m*)	0.0	90.0 (1.125)	460.0	2.667	13.500 (0.101)	24.000
*M*_*pacifica* → *sakhalina*_ (*N*_e_*m*)	0.0	3.3 (0.041)	413.3	0.000	7.167 (0.054)	15.333

## Discussion

Migratory birds may facilitate the spread of HPAI from Asia to North America (Winker and Gibson 2010). In this investigation, we used large sample sizes and two genetic data sources (mitochondrial DNA and microsatellites) to determine genetic structure patterns among six Dunlin subspecies that reside in and migrate through eastern Asia and North America. We specifically focused on determining whether the four subspecies of Dunlin that winter in Asia can be differentiated and if genetic evidence for gene flow among Beringian subspecies exists. We suggest that our results may be useful for documenting potential HPAI transmission routes and the pathways that may facilitate the spread of disease across continents.

Birds have reduced genetic structure relative to many other organisms, likely due to their capacity for flight and long distance movement (Greenwood and Harvey 1976; Zink et al. 1997). Many Arctic avian species, particularly migratory species, show lower levels of population genetic structure as a result of these high dispersal tendencies (Crochet 1996). For example, most shorebirds migrate long distances between breeding and nonbreeding areas (Brown et al. 2001), which may result in high gene flow and reduced genetic differentiation (e.g., Baker et al. 1994; Wenink et al. 1994; Haig et al. 1997; Wennerberg 2001b; Draheim et al. 2010; Miller et al. 2012). In contrast to past genetic studies of Dunlin that included limited sampling of Beringia-associated subspecies (e.g., Wenink et al. 1993; Wenink and Baker 1996; Wennerberg et al. 1999, 2008), genetic analyses from our investigation revealed marked genetic differentiation among some Dunlin subspecies based on mtDNA analyses. Phylogenetic analysis revealed four separate phylogroups with high levels of statistical support (Fig.2). Two of these groups consisted of samples from only *C. a. hudsonia* or *C. a. actites*, which occur in the most eastern and western regions of our study area. The other two groups contained mixtures of birds from more than one subspecies. The latter groups largely corresponded to birds that breed in relatively close proximity to one another, either in Asia (*C. a. sakhalina* and *C. a. kistchinski*) or in Alaska (*C. a. arcticola* and *C. a. pacifica*), although a few *C. a. sakhalina* birds from sites O and Q (Fig.1) possessed haplotypes from the *C. a. arcticola*/*C. a. pacifica* group (Fig.2). The lack of clear structure between the *C. a. sakhalina/kistchinski* and *C. a. arcticola*/*pacifica* groups suggests, in part, that the taxonomic status of these subspecies may require revision, although we recognize that other factors are important for defining subspecies (e.g., morphology, behavior, etc.; Haig et al. 2006).

Differentiation among subspecies was less pronounced based on the microsatellites, but significant structure was nonetheless detected between most subspecies pairs (Table3). Male-biased gene flow (Clark et al. 1997; Gibbs et al. 2000) or different evolutionary rates among markers (Brown 1983) are plausible hypotheses that may explain differences between data sets. However, adult male Dunlin usually exhibit higher breeding site fidelity relative to females (Soikkeli 1967, 1970; Jackson 1994; Tomkovich 1994; Hill 2012). Thus, the lower effective population size and greater strength of genetic drift associated with maternally inherited haploid genomes may be the most reasonable explanation for the greater differentiation identified in the mtDNA data. Regardless of data set, the genetic structure patterns that we detected are likely the result of some degree of breeding site fidelity (Warnock and Gill 1996; Hill 2012) and reasonably strong population-specific migratory connectivity exhibited by some subspecies (Fernández et al. 2008; Gill et al. 2013; S. Yezerinac and R. Lanctot, unpublished data).

Assuming that our sample of individuals and subspecies is representative of Dunlin from East Asia, our analysis suggests that we can use our data to obtain rudimentary estimates of the probability of correctly distinguishing Asian- versus Alaskan-breeding birds with mtDNA when sampling takes place in the East Asian nonbreeding areas. With the exception of seven *C. a. sakhalina* individuals, our representative mtDNA sequences from *C. a. sakhalina*,*C. a. kistchinski*, and *C. a. actites* (*n* = 107 total) were phylogenetically distinct from the haplotypes identified in Alaskan breeders (*C. a. arcticola*:*n* = 60; *C. a. pacifica*:*n* = 51; Fig.2). Thus, if an individual possessed a haplotype associated with the *C. a. actites* or *C. a. kistchinski*/*sakhalina* groups, the probability that the individual also breeds in Asia approaches 100% because no Alaska breeders possessed haplotypes from those groups. By contrast, if a bird sampled on the East Asia nonbreeding grounds possesses a haplotype from the main *C. a. arcticola*/*pacifica* group, our results suggest that the individual may either be *C. a. sakhalina* or *C. a. arcticola* (Fig.2; *C. a. pacifica* can be excluded from consideration given that this subspecies is entirely restricted to western North America). In this case, our application of Bayes' theorem indicates that there is nominally a ∽70% chance that a randomly selected bird possessing a haplotype from group I is *C. a. arcticola* (Table4). The probability of a correct inference becomes even larger as the population size ratio of *C. a. arcticola* to *C. a. sakhalina* increases (Table4). These probabilities are higher than the 53–60% correct assignment rates found by Gates et al. (2013) when using morphology to differentiate subspecies. Future analyses that combine genetic and morphological data may increase the likelihood of identifying *C. a. arcticola* in the Asian nonbreeding areas.

An unexpected outcome of our analyses included the detection of asymmetric gene flow from *C. a. arcticola* into *C. a. pacifica* and to a lesser extent also into *C. a. sakhalina* (Table5). After considering potential reasons for this pattern, we highlight the simple fact that *C. a. arcticola* performs the longest spring migration out of all of the subspecies examined and that its northbound migration pathway crosses over part of the *C. a. sakhalina* and *C. a. pacifica* breeding areas (Fig.1). It is feasible that some *C. a. arcticola* individuals ‘short-stop’ their migration in eastern Russia before crossing the Bering Sea to breed with *C. a. sakhalina,* and even more stop in western Alaska rather than continuing on to northern Alaska. Most reported cases of migratory short-stopping are associated with fall migrations *en route* to nonbreeding grounds, with the increased availability of supplemental food from agricultural systems (Wilson 1999; Jefferies et al. 2003) or climate change (Austin and Rehfisch 2005; La Sorte and Thompson 2007; Visser et al. 2009; Charmantier and Gienapp 2013) commonly invoked as possible explanations. In our case, we suggest that the frequency of short-stopping during spring migration may instead be correlated with poor weather conditions, resource limitations encountered during migration, or with the overall health and condition of the short-stopping individuals themselves. Evidence for migratory short-stopping during northbound breeding migrations has also been identified in lesser snow geese (*Chen caerulescens caerulescens*; Shorey et al. 2011). Given the shallow mitochondrial differentiation of *C. a. pacifica* and *C. a. arcticola* (Fig.2), we also cannot rule out the possibility that the signal of asymmetric gene flow is the result of recent divergence of the two subspecies. However, a recent divergence does not preclude the possibility of ongoing gene flow, especially considering the geographic proximity of the breeding ranges of the two subspecies, the long migration flight undertaken by *C. a. arcticola*, and the fact that the northbound migratory path leads directly over *C. a. pacifica*'s breeding range. In contrast, the signal of asymmetric gene flow from *C. a. arcticola* into *C. a. sakhalina* is most likely not the result of a recent divergence. The mtDNA-based phylogenetic tree illustrates that the two subspecies are reasonably well differentiated (Fig.2), thereby leaving gene flow as a more tenable explanation for the analysis outcome.

Our finding of asymmetric gene flow indicates that, in addition to *C. a. arcticola*'s usual northern Alaska breeding grounds, the western Alaska breeding grounds for *C. a. pacifica* need to be considered as a possible secondary entry point for Dunlin to carry AI into North America. This may be especially relevant if the migratory short-stopping behavior is influenced by an individual's health status, particularly if ill due to a viral disease. Because western Alaska and northern Alaska do not possess the same avian assemblages (Gabrielson and Lincoln 1959; Johnson and Herter 1989), the introduction of AI into western Alaska could lead to outbreaks in an additional and different suite of species than would an outbreak centered in northern Alaska.

The inference of asymmetric gene flow also implies the occurrence of direct interactions between *C. a. arcticola* and *C. a. pacifica* that could facilitate virus transmission between subspecies. Prior studies indicated that the two subspecies intermix during the fall after the breeding season (Taylor et al. 2011; Gill et al. 2013). If this were the only period of interaction, then the likelihood of HPAI spreading between subspecies would be low because any *C. a. arcticola* individuals harboring the virus would have had to (i) be infected on the wintering grounds and then (ii) live for 3–4 months with an active infection prior to intermixing with *C. a. pacifica* in the fall. However, our results suggest that individuals of the two subspecies sexually reproduce and thus likely share incubation duties for about 20 days (Warnock and Gill 1996). The breeding period occurs not long after migration and may coincide with the time when active shedding of HPAI by infected individuals is occurring.

Although our new findings do not specifically identify strategies for preventing the transmission of HPAI into North America, they nonetheless reveal a mechanism by which Dunlin could facilitate the spread of HPAI into North America and Mexico. This is particularly pertinent given that Dunlin are highly susceptible to infection with the H5N1 HPAI, and that some individuals may live to spread the disease, possibly after undergoing a migration (Hall et al. 2011). Although only a few Dunlin sampled in western North America have been documented with actively shedding AI (Ip et al. 2008; Iverson et al. 2008; USFWS and USGS 2011), the continued emergence of new HPAI strains (e.g., H5N8, H7N9) and the fact that most efforts to date have detected prior exposure (i.e., antibodies, see Pearce et al. 2012; Johnson et al. 2014) indicates that the evolution of new strains remains problematic and that Dunlin are a potential route for HPAI to reach and spread within North America.

## Data archiving statement

Data for this study are available at: GenBank Accession Numbers KP205084–KP205177 and KP205178–KP205271. Dryad Digital Repository http://dx.doi.org/10.5061/dryad.4t806.

## Appendix A

**Table:** Microsatellite and mitochondrial primer sequencers and PCR annealing temperatures (T_A_) used in Dunlin (*Calidris alpina*) analyses

	Primer names	Primer sequences	*T*_A_ (°C)
Microsatellites	CALP2R	5′-CAG AGC TGG AAG GT-3′	58
	CALP2F	5′-CAA AGG ATG TGG TT-3′	
	CME2R	5′-TTA AAA GGG ACC GAG TGT CCT-3′	58
	CME2F	5′-GGC TCT GCA TGA AAG TCT AAA TG-3′	
	CME10R	5′-TGT TAC CAA AGG CTT AAG CAA AG-3′	58
	CME10F	5′-GAA GGC GAG GAG AAC TTC TGT-3′	
	CME12R	5′-GTT GGG GGA CTA AAG GAA GAC-3′	58
	CME12F	5′-GAG CGG GAC GAG GAC AGT-3′	
	4A11R	5′-GGC ACA AAG CTC ACA CCT CTA TG-3′	58
	4A11F	5′-TCT AGC CTG AAA ATC TGT CCT TG-3′	
	D25R2	5′-CCT TGC TTT AGT CAA AGG TGA-3′	54
	D25F2	5′-GAG AGG ACC AGG AAA CAC T-3′	
	D26R	5′-GGA AGG CGT GTT GAT ACT G-3′	58
	D26F1	5′-CAG CGT GAC ATT AAC TCT CTG-3′	
	D110R1	5′-GAA ATT ACA AAG TAT GCT GAG-3′	54
	D110F1	5′-CAA CTA TAT CAG CAG GAA GCT-3′	
Cytochrome *b*	L14996	5′-AAY ATY TCW GYH TGA TGA AAY TTY GG-3′	55
	H15646	5′-GGN GTR AAG TTT TCT GGG TCN CC-3′	
Control region	TS 96L	5′-GCA TGT AAT TTG GGC ATT TTT TG-3′	53
	TS 778H	5′-AAA CAC TTG AAA CCG TCT CAT-3′	

## Appendix B

**Table:** Absolute and relative (in parentheses) frequencies of 94 combined mitochondrial cytochrome *b* and D-loop haplotypes within six Dunlin (*Calidris alpina*) subspecies

	Subspecies					
Haplotype	*arcticola*	*actites*	*hudsonia*	*kistchinski*	*pacifica*	*sakhalina*
H1	–	–	2 (0.125)	–	–	–
H2	–	–	1 (0.063)	–	–	–
H3	–	–	4 (0.250)	–	–	–
H4	–	–	2 (0.125)	–	–	–
H5	–	–	1 (0.063)	–	–	–
H6	–	–	1 (0.063)	–	–	–
H7	–	–	1 (0.063)	–	–	–
H8	–	–	3 (0.188)	–	–	–
H9	–	–	1 (0.063)	–	–	–
H10	1 (0.017)	–	–	–	–	–
H11	–	–	–	2 (0.067)	–	–
H12	–	–	–	5 (0.167)	–	–
H13	–	1 (0.043)	–	–	–	–
H14	–	1 (0.043)	–	–	–	–
H15	–	–	–	–	1 (0.020)	–
H16	–	2 (0.087)	–	–	–	–
H17	–	–	–	–	–	1 (0.019)
H18	–	–	–	6 (0.200)	–	9 (0167)
H19	–	–	–	–	–	1 (0.019)
H20	–	–	–	–	1 (0.020)	–
H21	–	–	–	1 (0.033)	–	–
H22	–	–	–	–	–	1 (0.019)
H23	–	–	–	1 (0.033)	–	–
H24	–	6 (0.261)	–	–	–	–
H25	–	1 (0.043)	–	–	–	–
H26	–	2 (0.087)	–	–	–	–
H27	–	–	–	–	–	1 (0.019)
H28	–	–	–	–	–	1 (0.019)
H29	–	1 (0.043)	–	–	–	–
H30	–	4 (0.174)	–	–	–	–
H31	–	5 (0.217)	–	–	–	–
H32	–	–	–	–	–	3 (0.056)
H33	–	–	–	1 (0.033)	–	–
H34	–	–	–	–	–	1 (0.019)
H35	–	–	–	–	–	1 (0.019)
H36	–	–	–	–	–	1 (0.019)
H37	–	–	–	–	–	1 (0.019)
H38	–	–	–	–	1 (0.020)	–
H39	–	–	–	1 (0.033)	–	–
H40	–	–	–	2 (0.067)	–	5 (0.093)
H41	–	–	–	–	1 (0.020)	
H42	–	–	–	–	10 (0.196)	
H43	–	–	–	–		1 (0.019)
H44	23 (0.383)	–	–	–	10 (0.196)	1 (0.019)
H45	1 (0.017)	–	–	–	–	–
H46		–	–	–	1 (0.020)	–
H47	2 (0.033)	–	–	–	3 (0.059)	–
H48	1 (0.017)	–	–	–	2 (0.039)	–
H49	1 (0.017)	–	–	–	–	–
H50	–	–	–	–	2 (0.039)	1 (0.019)
H51	–	–	–	–	1 (0.020)	–
H52	–	–	–	–	3 (0.059)	–
H53	–	–	–	1 (0.033)	–	–
H54	–	–	–	–	–	1 (0.019)
H55	–	–	–	1 (0.033)	–	–
H56	–	–	–	1 (0.033)	–	–
H57	–	–	–	–	–	1 (0.019)
H58	–	–	–	–	–	1 (0.019)
H59	1 (0.017)	–	–	–	–	–
H60	–	–	–	–	1 (0.020)	–
H61	–	–	–	–	–	1 (0.019)
H62	–	–	–	1 (0.033)	–	–
H63	–	–	–	–	–	1 (0.019)
H64	–	–	–	1 (0.033)	–	–
H65	–	–	–	1 (0.033)	–	–
H66	–	–	–	–	–	2 (0.019)
H67	1 (0.017)	–	–	–	–	–
H68	–	–	–	–	2 (0.039)	–
H69	–	–	–	3 (0.100)	–	5 (0.093)
H70	–	–	–	–	–	2 (0.037)
H71	–	–	–	–	–	1 (0.019)
H72	–	–	–	1 (0.033)	–	
H73	–	–	–	–	–	1 (0.019)
H74	–	–	–	–	–	1 (0.019)
H75	–	–	–	1 (0.033)	–	1 (0.019)
H76	–	–	–	–	–	1 (0.019)
H77	–	–	–	–	–	1 (0.019)
H78	9 (0.150)	–	–	–	8 (0.157)	4 (0.074)
H79	2 (0.033)	–	–	–	–	–
H80	4 (0.067)	–	–	–	–	–
H81	1 (0.017)	–	–	–	1 (0.020)	1 (0.019)
H82	1 (0.017)	–	–	–	–	–
H83	1 (0.017)	–	–	–	–	–
H84	3 (0.050)	–	–	–	–	–
H85	1 (0.017)	–	–	–	–	–
H86	1 (0.017)	–	–	–	–	–
H87	1 (0.017)	–	–	–	–	–
H88	2 (0.033)	–	–	–	–	–
H89	1 (0.017)	–	–	–	–	–
H90	1 (0.017)	–	–	–	–	–
H91	1 (0.017)	–	–	–	–	–
H92	–	–	–	–	1 (0.020)	–
H93	–	–	–	–	1 (0.020)	–
H94	–	–	–	–	1 (0.020)	–
Total	60	23	16	30	51	54

## Appendix C

Results from STRUCTURE analyses using eight microsatellite loci. The highest average likelihood was associated with the *K* = 5 case (panel A), suggesting the presence of five genetic clusters. However, assignment probabilities of individuals to these clusters were nearly uniform (panel B), indicating that *K* had been overestimated. Use of the Evanno et al. (2005) Δ*K* approach (panel C) suggested that there were two clusters; however, average assignment probabilities of individuals to these clusters were also uninformative (panel D). These results suggest that there is no detectable subspecies subdivision based on the microsatellites.

## Appendix D

**Table:** Allele frequencies from eight microsatellite loci within six Dunlin (*Calidris alpina*) subspecies

		Subspecies						
Locus	Allele size	*arcticola*	*actites*	*hudsonia*	*kistchinski*	*pacifica*	*sakhalina*	Overall
Calp2	120	0.0069	–	–	–	–	–	0.0027
	122	0.0278	–	–	0.0595	0.0132	0.0362	0.0270
	124	0.0174	0.2609	–	0.1190	0.0263	0.0942	0.0595
	126	0.2535	0.1957	0.1875	0.1071	0.1579	0.1594	0.1932
	128	0.1979	–	0.0625	0.0595	0.0921	0.0870	0.1216
	130	0.0486	–	0.0625	0.1071	0.1053	0.0580	0.0662
	132	0.0625	0.0652	0.0938	0.1667	0.0592	0.1087	0.0838
	134	0.0208	0.0435	0.1562	0.0595	0.0461	0.0580	0.0446
	136	0.1111	0.3696	0.1250	0.1429	0.0855	0.1667	0.1365
	138	0.2361	0.0652	0.3125	0.1548	0.3289	0.1377	0.2203
	140	0.0174	–	–	0.0119	0.0789	0.0725	0.0378
	142	–	–	–	0.0119	0.0066	0.0145	0.0054
	144	–	–	–	–	–	0.0072	0.0014
Cme2	137	0.0243	–	0.0312	–	0.0132	–	0.0135
	139	–	0.0870	–	–	0.0066	0.0145	0.0095
	141	0.0903	0.1739	–	0.0119	0.0395	0.0435	0.0635
	143	–	–	–	0.0357	–	–	0.0041
	145	0.0069	0.0652	–	–	0.0197	0.0290	0.0162
	147	0.1562	0.0217	0.0312	0.2024	0.0789	0.2101	0.1419
	149	0.2986	0.4783	0.0938	0.3452	0.3618	0.2464	0.3095
	151	0.2535	0.0652	0.2188	0.1548	0.2829	0.1739	0.2203
	153	0.0938	0.0217	–	0.1190	0.1316	0.0942	0.0959
	155	0.0590	–	0.4375	0.1071	0.0658	0.1014	0.0865
	157	0.0174	0.0870	0.1250	0.0238	–	0.0870	0.0365
	159	–	–	0.0312	–	–	–	0.0014
	163	–	–	0.0312	–	–	–	0.0014
Cme10	177	0.0035	–	–	–	0.0066	0.0072	0.0041
	181	0.9826	1.0000	0.9062	0.9881	0.9276	0.9565	0.9649
	183	0.0139	–	0.0938	0.0119	0.0658	0.0145	0.0270
	187	–	–	–	–	–	0.0217	0.0041
Cme12	164	–	–	–	0.0119	0.0066	–	0.0027
	166	0.0069	–	–	–	–	0.0145	0.0054
	168	0.0035	–	–	–	–	–	0.0014
	170	0.0278	0.0870	0.0312	0.0119	0.0329	0.0290	0.0311
	172	0.3229	0.1304	0.1562	0.3690	0.3816	0.3406	0.3243
	174	0.5174	0.3478	0.7500	0.4643	0.5000	0.5290	0.5095
	176	0.1215	0.4348	0.0625	0.1429	0.0789	0.0870	0.1257
4A11	139	–	–	–	–	0.0066	0.0072	0.0027
	141	0.2743	0.0870	0.2188	0.3452	0.3289	0.4130	0.3054
	143	0.6840	0.8696	0.7500	0.5833	0.6316	0.4928	0.6405
	145	0.0417	0.0435	0.0312	0.0714	0.0329	0.0870	0.0514
D25	326	0.0521	0.2391	0.0312	0.0714	0.0592	0.1594	0.0865
	328	0.8229	0.7609	0.5625	0.7381	0.8092	0.6957	0.7716
	330	0.0556	–	0.3750	0.1310	0.0329	0.0435	0.0676
	332	0.0694	–	0.0312	0.0476	0.0921	0.0870	0.0689
	334	–	–	–	0.0119	0.0066	0.0072	0.0041
	336	–	–	–	–	–	0.0072	0.0014
D26	237	0.0382	–	0.0312	0.0476	0.0461	0.0942	0.0486
	239	0.0104	–	–	–	–	–	0.0041
	241	–	–	–	–	–	0.0362	0.0068
	243	0.3993	0.5652	0.5938	0.3095	0.2500	0.4348	0.3838
	245	0.4167	0.1739	0.3750	0.4881	0.5592	0.3043	0.4162
	247	0.1111	0.2174	–	0.1429	0.0987	0.1159	0.1149
	249	–	0.0435	–	0.0119	0.0132	0.0072	0.0081
	251	0.0139	–	–	–	0.0263	0.0072	0.0122
	253	0.0104	–	–	–	0.0066	–	0.0054
D110	184	0.0729	–	0.0312	0.0238	0.0658	0.0290	0.0514
	186	0.1007	0.0217	0.1562	0.0833	0.1250	0.1159	0.1041
	188	0.3299	0.7826	0.5625	0.3452	0.2697	0.3188	0.3554
	190	0.4826	0.1957	0.2500	0.5119	0.5329	0.4928	0.4703
	192	0.0139	–	–	0.0357	0.0066	0.0362	0.0176
	196	–	–	–	–	–	0.0072	0.0014

## Appendix E

Posterior distributions for Bayesian estimates of mutation-scaled effective population sizes (*θ*) and migration rates (M) obtained from MIGRATE-N (Beerli and Palczewski 2010)

Point estimates and credibility intervals calculated from the posterior distributions are provided in Table5. See text for more details.

**mtDNA data:**
